# O.6.2-10 Community-based physical activity promotion programs in Porto metropolitan area: characterization and good-practices evaluation

**DOI:** 10.1093/eurpub/ckad133.280

**Published:** 2023-09-11

**Authors:** Beatriz França, Carolina Martins Machado, Sofia Franco, Carlos Pratas Valente, Romeu Mendes

**Affiliations:** Northern Regional Health Administration; Northern Regional Health Administration; National Physical Activity Promotion Program, Directorate-General of Health; Faculdade de Educação Física e Desporto, CIDEFES, Universidade Lusófona; Northern Regional Health Administration; National Physical Activity Promotion Program, Directorate-General of Health; Northern Regional Health Administration; EPIUnit - Instituto de Saúde Pública da Universidade do Porto; mbeatriz.santos@arsnorte.min-saude.pt

## Abstract

**Purpose:**

Physical activity (PA) is an important health determinant with recognised benefits. Community-based physical activity programs (CBPAP) are cost-effective in promoting PA. However, the number, type, and quality of these programs in Porto Metropolitan Area (PMA) are unknown. Therefore, the objective of this study was to identify, characterize and evaluate CBPAP in this geographic area.

**Methods:**

A systematic way to identify the ongoing CBPAP through the municipalities in PMA was defined. The interventions found were initially characterized through an online form. Then, the interventions that were CBPAP, according to this study criteria, were evaluated on a scale from 0-100% using a good practice characteristics appraisal tool developed by the Portuguese National Physical Activity Promotion Program. An estimated coverage of the programs, was calculated and the scores obtained in the evaluation tool were analysed.

**Results:**

Most municipalities acceded our request to participate (76,5%, n = 13), and 33 ongoing CBPAP were identified. Most of the programs do not restrict participants age (51,5%) and have no costs (75,8%). There were 14 modalities of activity being promoted, where multimodal training was the most common (45,5%). The utilization ratio of CBPAP in PMA was 640/100.000 inhabitants. The good practices evaluation revealed a mean score of 70,3% ± 12,6%. The questions about monitoring and evaluation scored lower (p < 0,001) and the questions about the program detailed characteristics scored higher (p < 0,001).

**Conclusions:**

The variety of exercise modalities and the large proportion of cost-free CBPAP are positive findings. Nonetheless, the low utilization ratio of these programs indicates they may not be a relevant PA promotion strategy in PMA. Clarification for which PA promotion strategies are implemented in this area may be needed, as well as determining if increasing the number of existing CBPAP, or to spread the existing ones, especially in the high population municipalities, are viable strategies. A failure in the evaluation processes of the existing programs was identified in this geographical area. Thus, and responding to national and international recommendations, in order to guarantee the effectiveness of the existing CBPAP, better monitoring and evaluation processes of programs should be implemented.

